# Work safety in food services

**DOI:** 10.47626/1679-4435-2023-1060

**Published:** 2023-11-24

**Authors:** Jéfferson Malveira Cavalcante

**Affiliations:** 1 Núcleo de Práticas Gastronômicas, Centro Universitário Christus - UNICHRISTUS, Fortaleza, CE, Brazil

**Keywords:** work accident, cuts, occupational illness, burns, posture, acidente de trabalho, cortes, doença do trabalho, queimaduras, postura

## Abstract

Numerous activities in food services can affect worker health and safety, given that daily food preparation routines involve the use of sharp instruments, steam, hot liquids, noise, frozen items, harsh cleaning chemicals, and heavy lifting. The objective of this study was to identify relationships among work activities that affect health and safety among food service workers. The methodology consisted of an integrative review of documentary and bibliographic research, using National Classification of Economic Activities as a data collection criterion and Ministry of Labor and Employment data on work accidents in food services between 2010 and 2020. Work accidents, which may or may not be accompanied by a work accident report, are classified according to type: at work, commuting to work, or occupational illness. During the study period, 169,609 work accidents occurred among food industry workers, which was 12% of the total number of work accidents recorded nationwide in all sectors. The most frequent accident types among food service workers were cuts, burns, falls, and those due to incorrect posture. Training, best practices, professional ethics, functional planning, and periodic and preventive maintenance are important strategies for providing the best working conditions in food services.

## Abstract (pt)

Os serviços de alimentação apresentam inúmeras atividades que se destacam na saúde e na preservação da integridade física dos colaboradores, pois no exercício laboral dentro do ambiente de preparação de alimentos, a utilização de instrumentos cortantes, vapor, líquidos quentes, ruídos, insumos congelados, manuseio de utensílios e equipamentos, contato com materiais de higienização, deslocamento e levantamento de peso fazem parte do cotidiano dos manipuladores. O objetivo da pesquisa foi identificar relações de atividades laborais na preservação da saúde e da integridade física de colaboradores em ambientes de trabalho que oferecem serviços de alimentação. A metodologia utilizada consistiu na revisão integrativa de pesquisas documental e bibliográfica, utilizando como critério de coleta de dados a Classificação Nacional de Atividades Econômicas e os acidentes que enquadram serviços de alimentação, ocorridos entre 2010 e 2020, disponibilizados pelo Ministério do Trabalho e Emprego. Os acidentes de trabalho são registrados de acordo com a Comunicação de Acidente de Trabalho em típico, de trajeto e doença do trabalho e sem Comunicação de Acidente de Trabalho. Foram registrados 169.609 acidentes de trabalho no ramo de alimentação, o que ocupa uma fatia de 12% no total de acidentes registrados no país. Nos serviços de alimentação, os acidentes que ocorrem com maior frequência são cortes, queimaduras, quedas e má postura de trabalho. A adoção de treinamento, boas práticas, ética profissional, planejamento físico-funcional, manutenção periódica e preventiva são as melhores alternativas para se propiciar as melhores condições nas atividades laborais, em serviços de alimentação.

## INTRODUCTION

The food service industry involves basic steps, such as purchasing, storing, preparing, and distributing food,^1^ and employee compliance with occupational safety rules is critical to ensure physical, mental, and social health. This can be accomplished through work activity training and surveillance,^2^ focusing on 3 aspects: engineering (periodic equipment maintenance), continuing education (strict safety policies for all procedures), and reinforcement (effective surveillance to avoid oversights and ensure compliance with established rules and procedures).^3^

Work activities in industrial kitchens include cleaning the work space and handling and preparing food, which entail repetitive movement of the upper limbs and spine, lifting weights, in addition to standing for long periods.^4^ The work conditions also include thermal discomfort, inadequate space, few breaks, and a fast pace, which can lead to repetitive strain injuries and work-related musculoskeletal disorders.^5^ The repetitive movement in kitchen work can be mitigated by investing in appropriate tools, thus avoiding musculoskeletal diseases.^6^

Occupational risks in food services include laceration injuries (from equipment such as knives or meat slicers), slips and falls (from wet floors or waste on the floor), and burns (from hot liquids, burners, ovens, or steam).

Good occupational health and safety practices are essential to prevent potentially dangerous situations related to planning failures, personal factors, or environmental conditions. These setbacks can be curtailed by improving each work activity or through collective and personal protective equipment, when relevant.^7^ From a holistic perspective, understanding the interconnection between environmental, sociocultural, biological, and psychological phenomena is the best way to reduce occupational risks in the work environment.^8^ The present study aimed to identify relationships between work activities and the health and well-being of employees in food services.

## METHODS

This exploratory study conducted an integrative review on occupational safety in food services through documentary and bibliographical research in: Google Scholar, SciELO, Minha Biblioteca (Group A), the CAPES Periodical Portal, and the Ministry of Labor and Employment (occupational health and safety statistical data).^9^

Given the growth and complexity of health data,^10^ integrative reviews focus on scientific evidence, synthesizing results in a systematic, orderly, and comprehensive way, leading to a more complete understanding of the topic of interest.^11^

In relation to the research data (Table 1), the National Classification of Economic Activities (Classificação Nacional de Atividades Econômicas: CNAE) was selected as a parameter for bibliographic data related to work accidents and occupational illnesses among food service workers from 2010 to 2020.

**Table 1:** Economic classification of Brazilian food services

CNAE	Economic activity
56.11-2	Restaurants and other food and beverage service establishments
56.12-1	Mobile food services
56.20-1	Catering, buffets, and other prepared food services

Source: National Classification Commission/ Brazilian Institute of Geography and Statistics (Comissão Nacional de Classificação/ Instituto Brasileiro de Geografia e Estatística).^12^

CNAE = National Classification of Economic Activities (Classificação Nacional de Atividades Econômicas).

## RESULTS AND DISCUSSION

Work accidents are defined as uncertain, undesirable, and improbable events resulting from complex interactions between physical, biological, psychological, social, and cultural factors.^13^ Work accidents and occupational illnesses, which are caused by unsafe acts and/or unsafe conditions,^14^ are a constant organizational and governmental concern worldwide.

Work accidents in Brazil are documented by the Ministry of Labor and Employment (Ministério do Trabalho e Emprego), with support from the Brazilian Institute of Geography and Statistics (Instituto Brasileiro de Geografia e Estatística). They are entered into the database with or without an accompanying work accident report and are classified as accidents that occur at work, while commuting to work, or as occupational disease/illness.

Between 2010 and 2020, 169,609 accidents occurred among food service industry workers (Table 2), which was 12% of the total number of work accidents nationwide among all work activities.

**Table 2:** Work accidents in Brazilian food services

CNAE	Accidents with WAR			Accidents without WAR	Total
	At work	Commuting	Occupational disease		
56.11-2	65,223	23,331	1703	19,390	109,647
56.12-1	392	130	14	203	739
56.20-1	37,256	8,988	1097	11,882	59,223

Source: Ministry of Labor and Employment (Ministério do Trabalho e Emprego) data.

56.11-2 = Restaurants and other food and beverage service establishments; 56.12-1 = Mobile food services; 56.20-1 = Catering, buffet, and other prepared food services; WAR = Work Accident Report; CNAE = National Classification of Economic Activities (Classificação Nacional de Atividades Econômicas).

Among the 3 included economic activity codes, approximately 110,000 (65%) of the accidents between 2010 and 2020 occurred in restaurants and other food and beverage establishments (CNAE 51.11-2)(Table 2).

In Brazil, work accident reports are mandatory, even in cases when no sick leave is required. An ordinance by the Ministry of Economy’s Special Secretariat for Social Security and Labor (Secretaria Especial de Previdência e Trabalho, Ministério da Economia - SEPRT/ME No. 4334/2021) stipulates the procedures and data required for work accident reports: the employee’s government identification number, employer data, description of the accident, classification as accident or illness/disease, medical care records, description of the injury, diagnosis, and observations.^15^

**Figure 1:**
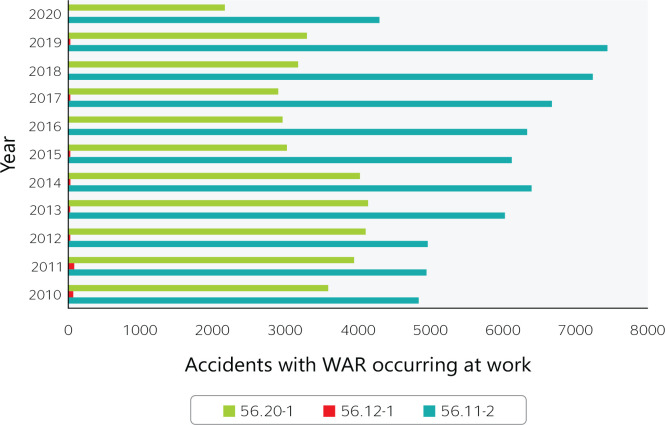
Accidents with a work accident report (WAR) occurring at work in Brazilian food services from 2010 to 2020. 56.11-2 = Restaurants and other food and beverage service establishments; 56.12-1 = Mobile food services; 56.20-1 = Catering, buffet, and other prepared food services. Source: Ministry of Labor and Employment (Ministério do Trabalho e Emprego) data.

Typically, work accidents result from work activities or conditions,^16^ eg, cuts, falls, burns, and trauma,^17^ which are the most common accident types in food preparation environments. Between 2010 and 2020, more than 100,000 work accident reports were filed by Brazilian food services (Figure 1), representing approximately 7% of the total number of work accidents in all sectors nationwide. Typical procedures/factors involved in accidents include: cutting plant and animal-based foods, cooking, working posture, and organizing the workspace.

**Figure 2:**
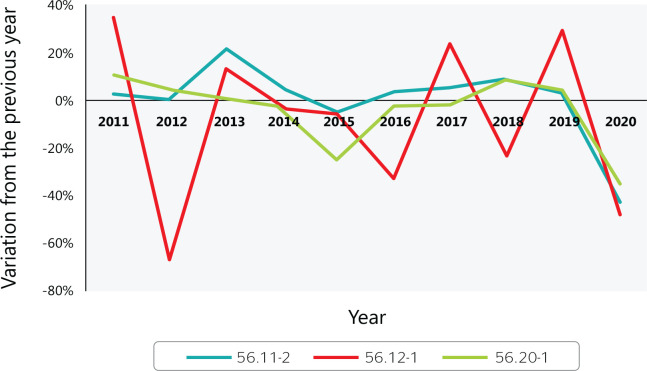
Variation in accidents at work with a work accident report in Brazilian food services from 2010 to 2020. 56.11-2 = Restaurants and other food and beverage service establishments; 56.12-1 = Mobile food services; 56.20-1 = Catering, buffet, and other prepared food services. Source: Ministry of Labor and Employment (Ministério do Trabalho e Emprego) data.

Figure 2 shows the year-to-year fluctuation in accidents between 2010 and 2020 among food service workers while at work, which was affected by the use of personal protective equipment, training, and caution to avoid cuts, burns, and falls.

**Figure 3:**
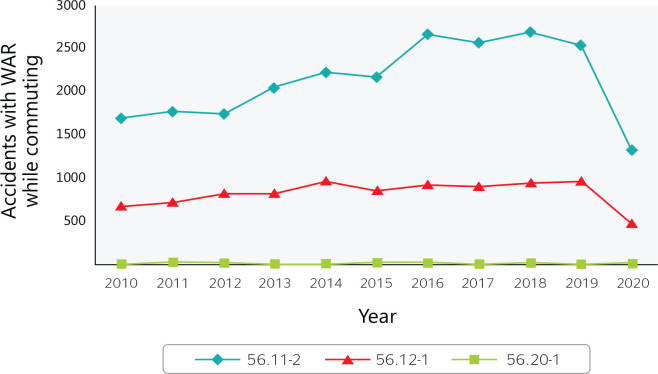
Accidents with a work accident report (WAR) that occurred while commuting to work in Brazilian food services from 2010 to 2020. 56.11-2 = Restaurants and other food and beverage service establishments; 56.12-1 = Mobile food services; 56.20-1 = Catering, buffet, and other prepared food services. Source: Ministry of Labor and Employment (Ministério do Trabalho e Emprego) data.

Between 2010 and 2020, approximately 2% (32,000) of the reported accidents while commuting to work in Brazil occurred among food service workers (Figure 3). Except for mobile food services, there was a notable decrease in 2020, reflecting the COVID-19 pandemic lockdown.

**Figure 4:**
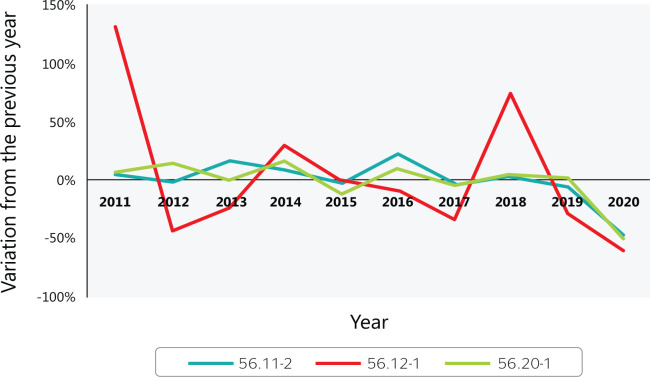
Variation in work accidents (with a work accident report) while commuting to food services in Brazil from 2010 to 2020. 56.11-2 = Restaurants and other food and beverage service establishments; 56.12-1 = Mobile food services; 56.20-1 = Catering, buffet, and other prepared food services. Source: Ministry of Labor and Employment (Ministério do Trabalho e Emprego) data.

Figure 4 shows the year-to-year variation in accidents that occurred while commuting to work among food service workers. The high variation in mobile food services between 2010-2012 and 2017-2019 are due to a small number of accidents during a given year (Figure 3).

Government intervention through education and improved road infrastructure, combined with employer investment in transportation (eg, chartering vans or buses for employees)^18^ and defensive driving by employees, can reduce road accidents for any type of economic activity.

**Figure 5:**
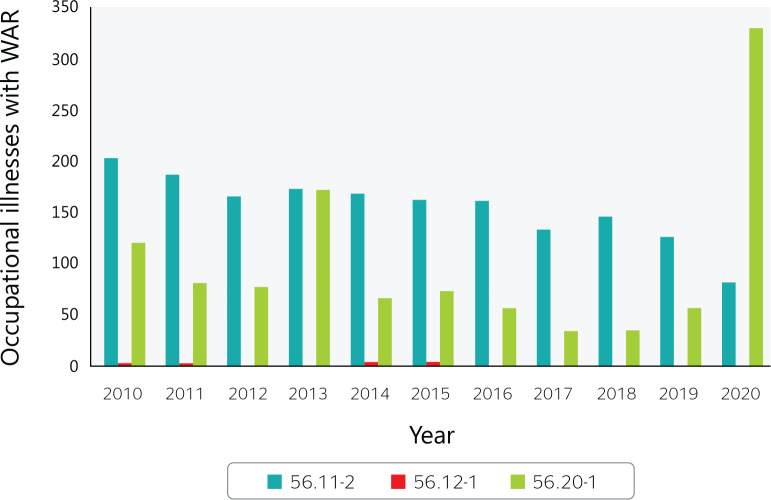
Occupational illnesses with a work accident report (WAR) in Brazilian food services from 2010 to 2020. 56.11-2 = Restaurants and other food and beverage service establishments; 56.12-1 = Mobile food services; 56.20-1 = Catering, buffet, and other prepared food services. Source: Ministry of Labor and Employment (Ministério do Trabalho e Emprego) data.

Between 2010 and 2020, 2814 occupational illnesses were reported among food service workers, representing 0.2% of all occurrences nationwide (Figure 5).

**Figure 6:**
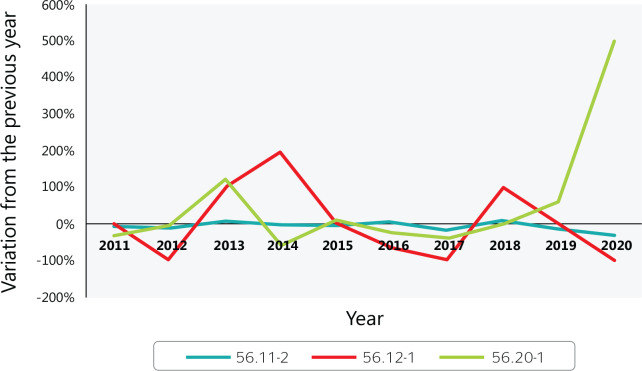
Variation in reported occupational illness in Brazilian food services from 2010 to 2020. 56.11-2 = Restaurants and other food and beverage service establishments; 56.12-1 = Mobile food services; 56.20-1 = Catering, buffet, and other prepared food services. Source: Ministry of Labor and Employment (Ministério do Trabalho e Emprego) data.

In 2020, 330 reports of occupation illness occurred among catering, buffet and other prepared food service workers (CNAE 5620), an increase of 80% (Figure 6) over 2019. According to Ayres & Corrêa,^16^ The most common occupational work-related musculoskeletal disorders are:

- Tendinitis - inflammation of the shoulder, elbow, and wrist;
- Low back pain - pain in the lumbar region;
- Myalgia - muscle pain in different parts of the body.

Workplace exercise programs tend to increase productivity and reduce costs due to sick leave, promoting health in the workplace.^19^

**Figure 7:**
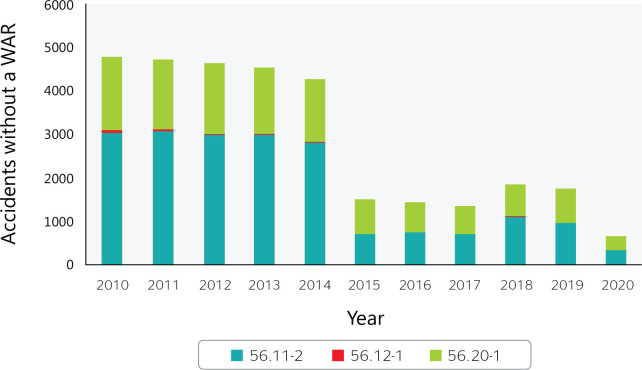
Work accidents without a work accident report (WAR) in Brazilian food services from 2010 to 2020. 56.11-2 = Restaurants and other food and beverage service establishments; 56.12-1 = Mobile food services; 56.20-1 = Catering, buffet, and other prepared food services. Source: Ministry of Labor and Employment (Ministério do Trabalho e Emprego) data.

A total of 31,475 work accidents without work accident reports occurred in Brazilian food services between 2010 and 2020 (Figure 7), representing 2.3% of all accidents. In 2010, there were 4770 work accidents without work accident reports, while in 2020 this number had dropped to 652, a reduction of 86%, which reflects greater employer concern to conform to the law.

Current Brazilian legislation requires issuance of a work accident report, regardless of the accident’s severity or consequence, which must be filed on the first business day after the accident or immediately in accidents resulting in death. Failure to file work accident reports, which is punishable by fines, directly affects planning, reporting, and control by the National Social Security Institute (Instituto Nacional do Seguro Social) and Social Security agencies.

**Figure 8:**
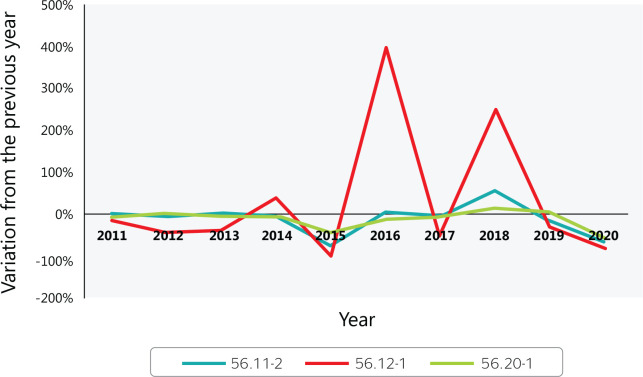
Year-to-year variation in accidents without a work accident report in Brazilian food services from 2010 to 2020. 56.11-2 = Restaurants and other food and beverage service establishments; 56.12-1 = Mobile food services; 56.20-1 = Catering, buffet, and other prepared food services. Source: Ministry of Labor and Employment (Ministério do Trabalho e Emprego) data.

Figure 8 shows the variation in food service accidents without work accident reports from 2010 to 2020. Most between-year variations were small, except for 2016-2015 and 2018-2017 in mobile food services (CNAE 5612), in which the rate increased significantly and alarmingly.

According to CNAE commerce/accommodation/food records for the state of Bahia in 2000, work accidents led to many lost days and had a great impact on productivity due to temporary work incapacity. Moreover, many of these injuries were considered avoidable, which reinforces the need for preventive actions to reduce Social Security costs.^20^ This corroborates the need for worker health initiatives, in addition to greater investment in intervention and longitudinal research in economic activity in Brazil and worldwide.^21^

Casarotto & Mendes^4^ studied 4 university restaurants and a university hospital kitchen, identifying ergonomics as the reason for the high number of occupational injuries (low back pain) and accidents (cuts and burns). Workplace safety evidence from a survey of another university restaurant indicated that ergonomic and psychosocial risks negatively affected the mental and physical health of employees.^22^

Food service environments involve a series of physical risks, including lighting, noise, temperature, and ergonomic issues.^23^ However, worker commitment to food safety is also critical, for example complying with standardized operating procedures in the event of respiratory, gastrointestinal symptoms, skin lesions, or any other sign of illness.^24^

A study of 93 food handlers in a food service in Juiz de Fora, Minas Gerais, found that training in personal protective equipment use, especially continuing education, was important to ensure worker protection,^25^ given that inadequate infrastructure and incorrect use of personal protective equipment increase the risk of accidents.^26-29^

In an epidemiological study, Konishi et al.^30^ found a series of occupational dermatosis (warts) cases among supermarket employees in the city of São Paulo, probably due to sharing work instruments, which highlights the importance of reviewing and adapting procedures to guarantee satisfactory occupational hygiene.

Interventions in the organizational and psychosocial aspects of work, including strategies to minimize tiredness, discouragement, and discontent^31,32^ in industrial and commercial kitchens,^5^ must be implemented to improve employee safety, health, and comfort.^33^ Studies by Castro & Okawa^34^ and Lacerda et al.^35^ on workplace safety in the food service industry found a lack of effective action by the Internal Accident Prevention Commission (Comissão Interna de Prevenção de Acidentes). In food services, functional planning of the work environment must expedite continuous flow, operability, sustainability, and ergonomic working conditions, since the workspace is full of environmental (physical, chemical, and biological) and ergonomic hazards. Reported accidents are identified according to intensity in risk maps prepared by Internal Accident Prevention Commission members.

When implemented, preventive projects such as the Environmental Risk Prevention Program (Programa de Prevenção de Riscos Ambientais) and the Medical Control and Occupational Health Program (Programa de Controle Médico e Saúde Ocupacional) can increase occupational health and safety by contributing to the development and improvement of inspection procedures and research instruments.^17^

## CONCLUSIONS

These accident data indicate the need for better working conditions in the food service industry to avoid, in so far as possible, all sorts of accidents, as well as the need for a greater commitment to occupational safety by employers and employees. Training, best practices, professional ethics, functional planning, ergonomic postures, the correct use of personal protective equipment, and preventive maintenance are important strategies for achieving the best food service work conditions, since a commitment to ensuring health and preserving physical integrity must always be prioritized.
